# Optical coherence elastography detects increased corneal stiffness in nonhuman primates with experimental glaucoma

**DOI:** 10.1117/1.JBO.30.12.124508

**Published:** 2025-10-11

**Authors:** Amandeep Singh, Achuth Nair, Zhihui She, Mohammed Dehshiri, Manmohan Singh, Salavat Aglyamov, Nimesh Patel, Kirill Larin

**Affiliations:** a University of Houston, Department of Biomedical Engineering, Houston, Texas, United States; b University of Houston, College of Optometry, Houston, Texas, United States

**Keywords:** cornea, shear wave imaging, optical coherence elastography, experimental glaucoma

## Abstract

**Significance:**

Glaucoma is a leading cause of irreversible blindness, characterized by progressive optic nerve damage. Early detection of glaucoma is key to effective intervention, but an incomplete clinical understanding of the development of glaucoma complicates the selection of diagnostic criteria. Prolonged ocular hypertension due to glaucoma can impact the biomechanical properties of ocular tissues, including the cornea. We examine whether experimental glaucoma causes changes in the biomechanical properties of the cornea.

**Aim:**

We determined the biomechanical properties of the cornea in a nonhuman primate model of unilateral experimental glaucoma and compared them with the fellow, untreated control eyes using optical coherence elastography (OCE) to determine if prolonged intraocular pressure (IOP) elevation causes changes in corneal stiffness.

**Approach:**

Experimental glaucoma was induced in one eye (*Macaca mulatta*, N=2) by lasering the trabecular meshwork, whereas the fellow eye was used as a control. Both eyes were imaged with wave-based OCE to investigate the inter-ocular difference in stiffness. Measurements were taken at three different frequencies with quasi-harmonic excitation, and central corneal thickness was measured along with IOP in each eye.

**Results:**

Our results show a significant (p<0.01) increase in wave speed in the experimental glaucoma eye compared with the control eye for both subjects.

**Conclusions:**

These results show the potential of wave-based OCE methods for assessing stiffness changes in the cornea caused by remodeling due to chronic pressure elevation.

## 1 Introduction

Glaucoma describes a group of diseases that is known to affect over 80 million people worldwide.^1^^,^^2^ The disease is multifactorial, characterized by the death of retinal ganglion cells and remodeling of connective tissues at the optic nerve head.^3^ Glaucoma is a unique optic neuropathy, and the cellular and structural processes related to its development and pathogenesis are not well understood. One of the primary risk factors for the development and progression of primary open-angle glaucoma (POAG) is ocular hypertension, or raised intraocular pressure (IOP).^4^^,^^5^ Prolonged IOP elevation is known to cause structural and functional changes to both neural and connective ocular tissues. Alongside tests to evaluate visual function and screening to monitor IOP, various optical imaging modalities, including optical coherence tomography (OCT), have been the primary means for diagnosing glaucoma and staging its progression.^6^ However, these approaches are largely based on detecting structural and functional changes to ocular structures after symptoms of glaucoma have developed. Furthermore, there is a paucity of methods for the noninvasive assessment of the unique connective tissue remodeling known to occur due to the development and progression of glaucoma.

Glaucoma treatment is limited to IOP reduction, either with pharmacological or surgical methods. Although effective in both high and normal tension disease, pressure reduction itself is often insufficient for halting disease progression.^7^ There is a need to identify and target the underlying disease mechanisms to effectively diagnose and develop treatments for glaucoma.

To that end, various studies have examined changes in ocular tissue structure during the development of glaucoma down to the cellular level. Studies suggested that the pathogenesis of glaucoma may cause stiffening in the trabecular meshwork (TM) and the lamina cribrosa (LC) within the optic nerve head (ONH).^8^^–^^10^ The mechanical properties of these structures are known to be regulated by extracellular matrix composition, including the distribution of structural proteins such as collagen. Furthermore, studies showed an increase in stiffness in these tissues due to the nonenzymatic crosslinking between collagen molecules.^11^^,^^12^ The ECM of the corneal stroma includes collagen types I, II, and V, glycosaminoglycans (GAGs), and proteoglycans, which are similar to the ECM of the TM and the ONH. Although there are several differences between the structural components of these tissues, these similarities suggest that there may be some similarity in remodeling mechanisms.

The Ocular Hypertension Trial Study identified that thinner corneas could predict the development of POAG, and various studies suggested that reduced corneal hysteresis (suggesting a stiffer cornea) could be a factor in the development and progression of glaucoma.^5^^,^^13^ Changes in corneal geometry and stiffness observed in glaucoma, given the cornea’s similar structural protein composition, suggest that the ECM remodeling responsible for altered stiffness in the ONH and TM may also occur in the cornea. However, most clinically available techniques for measuring corneal stiffness, including the ORA and the Corvis ST, are limited by the inherent dependence of measured stiffness, thickness, and IOP, so separating corneal stiffness from IOP and thickness may not be feasible using these techniques.

Elastography presents an alternative approach for measuring the biomechanical properties of tissues and has been used extensively for measuring ocular biomechanics.^14^ Ultrasound elastography has been previously used to measure tissue stiffness in human subjects.^15^ However, this approach requires physical contact with the eye, and its parent imaging modality provides limited spatial resolution and contrast in ocular tissues, potentially limiting its utility as a tool for early glaucoma detection.^16^^,^^17^ Brillouin microscopy^18^^,^^19^ has recently been utilized for measuring corneal biomechanical properties in human subjects, but there are limitations in acquisition time and spatial sampling. Furthermore, translating the Brillouin frequency shift to Young’s modulus is an ongoing area of investigation.^20^^,^^21^

Optical coherence elastography (OCE),^22^ a functional extension of OCT,^23^ has been recently implemented in various clinical studies for measuring the biomechanical properties of the cornea, as well as several other tissues.^24^^,^^25^ OCE takes advantage of the superior spatial resolution and displacement sensitivity of OCT to perform high-resolution, noncontact assessment of tissue biomechanical properties.^25^^–^^27^ The most common approach for OCE of the cornea is wave-based elastography.^28^ Briefly, an elastic wave is induced in the cornea, and the propagation of this elastic wave is detected using OCT.^24^^,^^27^ The speed of the propagating wave can be directly related to Young’s modulus, enabling a rapid, high-resolution, noncontact approach for measuring corneal stiffness. Various wave-based OCE approaches have been developed, each with unique advantages and limitations. These approaches include air-pulse,^29^ acoustic radiation force,^30^ contact probes,^31^ and air-coupled ultrasound.^32^^–^^34^ Various implementations of wave-based OCE have been demonstrated for a variety of applications in developmental biology,^35^ ophthalmology,^36^ and dermatology.^37^^,^^38^

In this work, we utilized an OCE approach to measure the biomechanical properties of the cornea in a nonhuman primate (NHP) model of unilateral experimental glaucoma. OCE measurements were taken on the cornea of each eye to measure corneal stiffness after exposure to glaucoma-induced changes in corneal stiffness. The sinusoidal excitations of different frequencies were delivered using a blunt needle attached to a piezoelectric actuator, and wave propagation was imaged with a phase-sensitive OCT system. The particle velocity algorithm was used to reconstruct the space-time map, and linear regression was used to calculate the speed of wave propagation.

## 2 Materials and Methods

### 2.1 Subject Preparation

For this pilot study, two rhesus monkeys (*Macaca mulatta*) were examined (aged 3.8 and 7.9 years). Each subject had experimental glaucoma induced in the right eye, and the left eye served as a control. For the experimental eye, the TM was scarred using contiguous burns from a 532 nm diode laser (Zeiss Visulas 532; Carl Zeiss Meditec, Jena, Germany) focused on with a Kaufman single mirror gonioscopy lens (Ocular Instruments, Bellevue, Washington, United States). Ablation parameters include a nominal power range from 800 to 1000 mW, a 50  μm spot size, and an application duration of 500 ms. Laser power was adjusted for optimal blanching of the TM tissue, to minimize “popping” and bleeding, thereby ensuring controlled and consistent tissue response. The initial treatment procedure involves lasering 180 deg of the angle, followed by 90 deg treatments at two-to-three-week intervals until sustained IOP elevation consistent with glaucoma is achieved. Further details of the ablation procedure can be found in previous work.^39^

For the imaging procedure, the animals were anesthetized with an intramuscular injection of ketamine (20 to 25 mg/kg/h) and xylazine (0.8 to 0.9 mg/kg/h) to ensure adequate sedation. In addition, a subcutaneous injection of atropine sulfate (0.04 mg/kg) was administered to reduce salivary secretions and help maintain a stable, healthy heart rate throughout the procedure. Each animal was placed in a prone position with the head facing forward and oriented using a bite bar. A speculum was used to hold the eyelids open, and the eyes were hydrated using saline when not being imaged. Prior to OCE imaging, IOP was measured using a tonometer (iCare TONOVET plus) in both subjects. Measurements were taken at two time points: immediately after sedation, reflecting the normal, diurnal intraocular pressure, and just before the OCE imaging procedure, after the subject had been anesthetized for more than 1 h and IOP had reduced due to the pressure-depressive effects of sedation.^40^ All experimental procedures and animal care protocols were approved by the Institutional Animal Care and Use Committee (IACUC) at the University of Houston. The use of animals in this study adhered strictly to the National Institutes of Health (NIH) Guidelines for the Care and Use of Laboratory Animals.

### 2.2 Experimental Setup and Data Acquisition

**Fig. 1:**
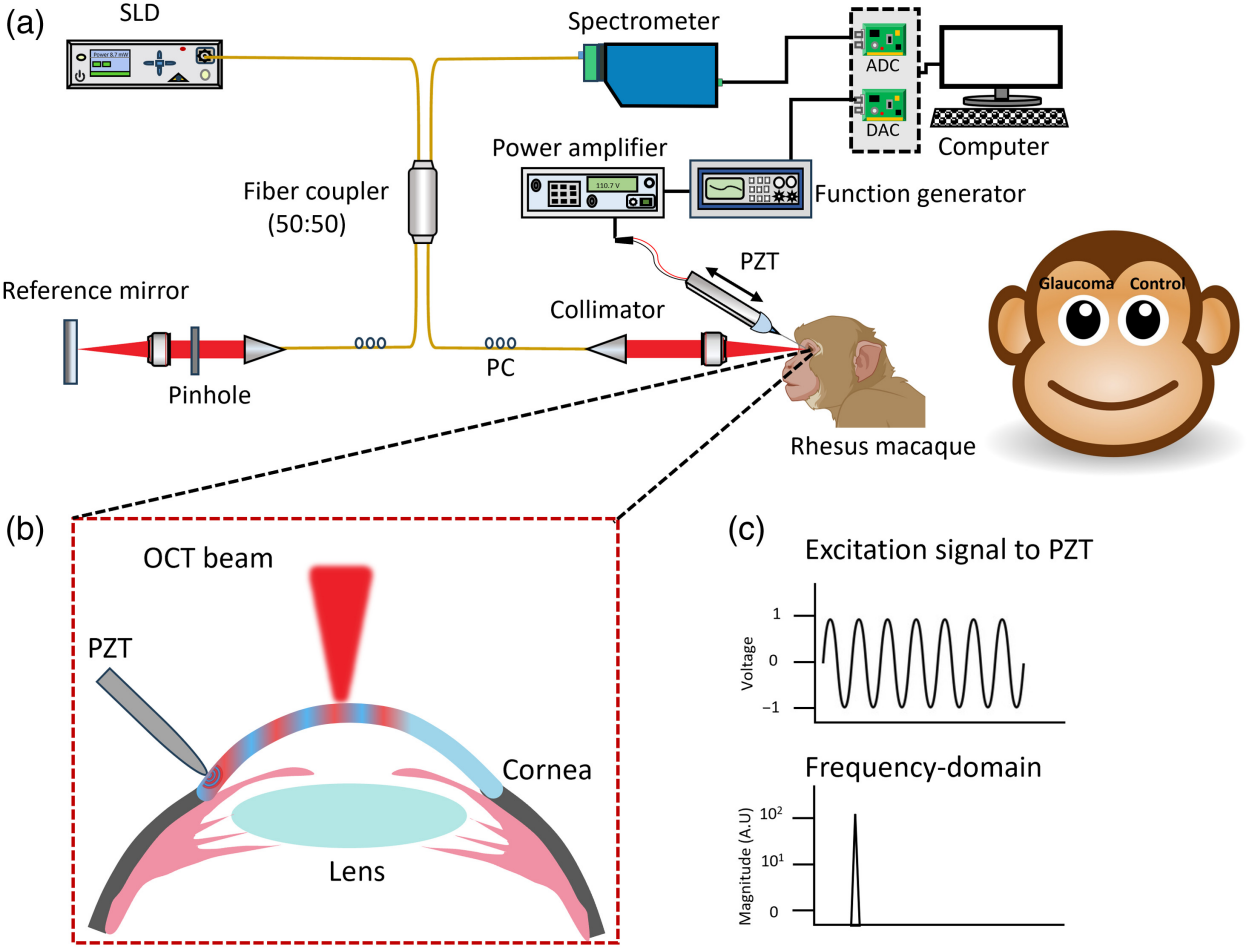
(a) OCE setup for biomechanical measurements. ADC, analog to digital converter; DAC, digital to analog converter; PC, polarization control; PZT, piezoelectric actuator; SLD, superluminescent diode. (b) The inset shows mechanical excitation and wave propagation in the cornea. (c) Representation of the sinusoidal excitation signal and its frequency content.

A schematic illustration of the OCE setup is presented in Fig. 1(a). The system consists of a phase-sensitive optical coherence tomography (PhS-OCT) platform that incorporates a broadband superluminescent diode (S840-B-I-20; Superlum Diodes Ltd., Carrigtwohill, Ireland), having a central wavelength of 840 nm with a spectral bandwidth of ∼40  nm. The axial and lateral resolutions were ∼9.7 and ∼6.2  μm, in air. The displacement stability was <1  nm at an OCT signal-to-noise ratio >30  dB. OCE measurements were performed in M-B-mode at a camera rate of 50 kHz.^27^^,^^41^ A blunt gavage needle was attached to a piezoelectric actuator (SA030318, PiezoDrive, Australia) to induce mechanical perturbation at the temporal limbus. The needle was placed in contact with the eye at an angle of incidence of ∼45  deg to the limbal surface. A function generator (DG4162, RIGOL Tech.) produced a sinusoidal signal of different excitation frequencies (1, 3, and 5 kHz), and an amplifier (MX200, PiezoDrive, Australia) was used to amplify the signal before driving the actuator.

Wave propagation across the cornea was detected using an M-B mode imaging approach scanning in a temporal-nasal orientation. Each M-mode scan consisted of 1000 temporal A-lines (20 ms) acquired at each of 500 spatial lateral points (∼7.7  mm). Wave speed estimation using this approach is described in detail in our previous works.^33^^,^^41^ Briefly, wave propagation across the cornea is detected based on the temporal phase difference at each sampled point. Equation (1) represents how the phase difference is translated to axial particle velocity

$$v_{z}(t)=\frac{\lambda_{0}}{4\pi n\tau }\Delta \phi_{z}(t),$$ (1)

where $\lambda_{0}=840\;\mathrm{nm}$ was the central wavelength of the light source, n was the refractive index of the tissue sample, τ=20  μs was the time interval, and $\Delta \phi_{z}(t)$ was the phase difference between consecutive A-lines. A finite impulse response (FIR) filter was applied to the axial particle velocity signal at each excitation frequency (1, 3, and 5 kHz) using a 200 Hz bandwidth. OCT images acquired during OCE imaging were used to estimate central corneal thickness (CCT) from the top and bottom surfaces of the apex of the cornea after accounting for the refractive index.

Statistical analysis was conducted to observe underlying trends and the level of significance of wave speeds between the control and experimental glaucoma cornea at each excitation frequency. We performed Mann–Whitney U-tests to compare the differences in wave speed for both types of corneas (control and experimental glaucoma). A p-value of <0.05 was assumed to be statistically significant for all analyses.

## 3 Results

**Fig. 2:**
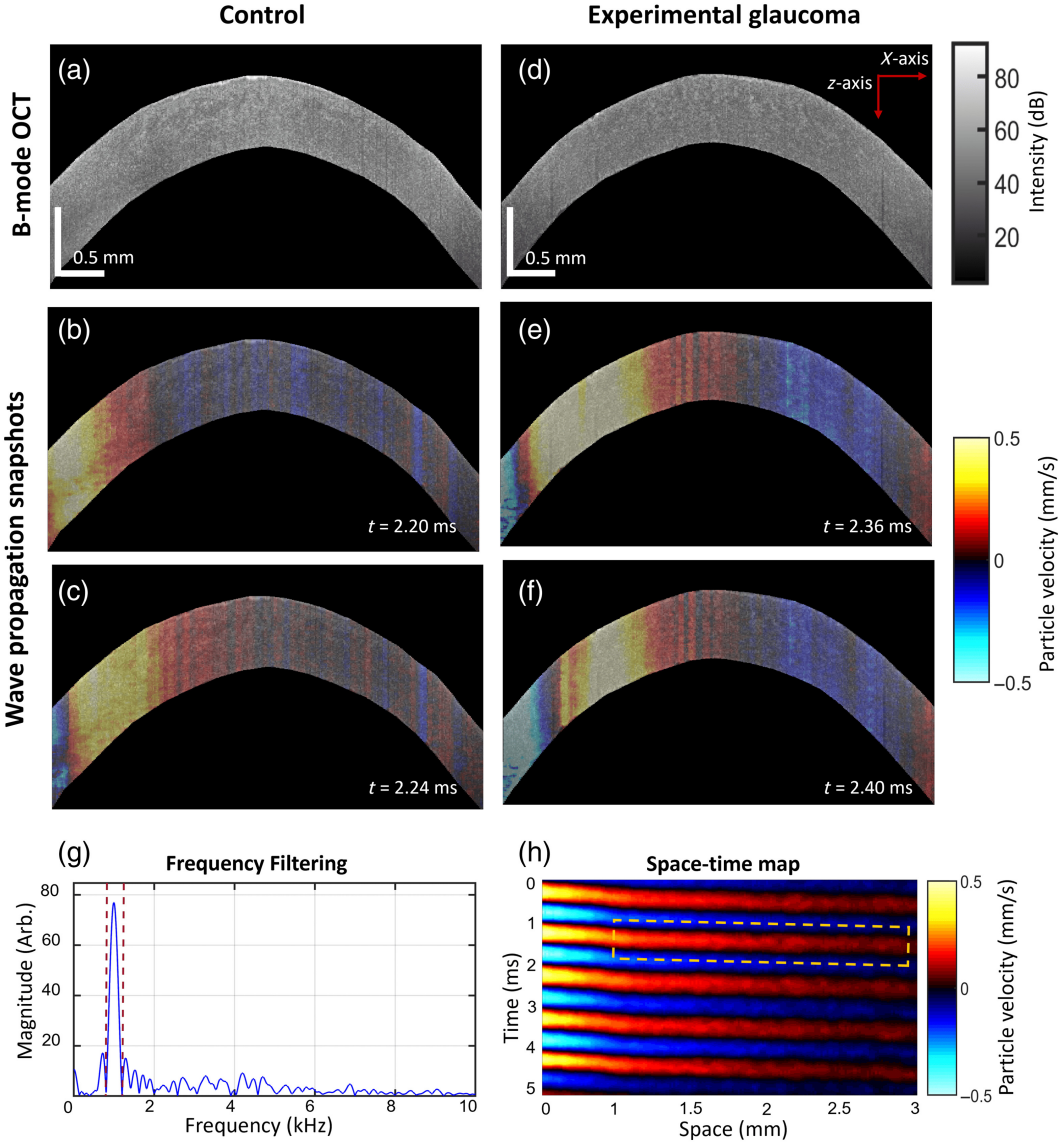
(a) B-mode OCT image of control cornea. Snapshots of the wave propagation at time (b) t=2.20  ms and (c) t=2.24  ms after excitation. (d) B-mode OCT image of the cornea with experimental glaucoma procedure. Snapshots of the wave propagation at time (e) t=2.36  ms and (f) t=2.40  ms after excitation. (g) The spectrum of the axial particle velocity, where the red dashed lines are the lower and upper bounds used for filtering. (h) An example of a space-time map of the wave propagation on the cornea.

Figures 2(a) and 2(d) are cross-sectional B-mode OCT images of the cornea from a control eye and an experimental glaucomatous eye, respectively. Figures 2(b) and 2(c) illustrate example time snapshots (t=2.20  ms and t=2.24  ms) capturing the propagation of mechanically induced elastic waves in the control cornea at 3 kHz. Figures 2(e) and 2(f) show sequential snapshots at time points t=2.20  ms and t=2.24  ms, respectively, showing the wave propagation in the cornea at 3 kHz for the experimental glaucomatous eye. Figure 2(g) represents an example of the frequency spectrum of the axial particle velocity. The red dashed lines are the high-pass and low-pass cutoff frequencies applied during temporal filtering to isolate the relevant frequency band for wave analysis. Figure 2(h) shows an example of a space-time map for the wave propagation, and the yellow dotted rectangle highlights the region of interest used to calculate wave speed that excludes the near field region.

The quantitative measurements for CCT and IOP are tabulated in Table 1. The CCT measured prior to OCE imaging for both (control and experimental glaucoma) eyes showed that the treated eye had a thinner cornea, albeit only very slightly, and both glaucoma eyes in both subjects had elevated IOP immediately post-sedation but normalized prior to OCE measurement.

**Table 1:** CCT and IOP of NHP subjects.

	CCT (μm)		Immediate post-sedation IOP (mmHg)		Pre-OCE measurement IOP (mmHg)	
	Control	Glaucoma	Control	Glaucoma	Control	Glaucoma
Subject 1	423.7±3.9	418.7±1.6	15	28	11	12
Subject 2	481.5±1.2	469.1±1.6	10	24	6	8

**Fig. 3:**
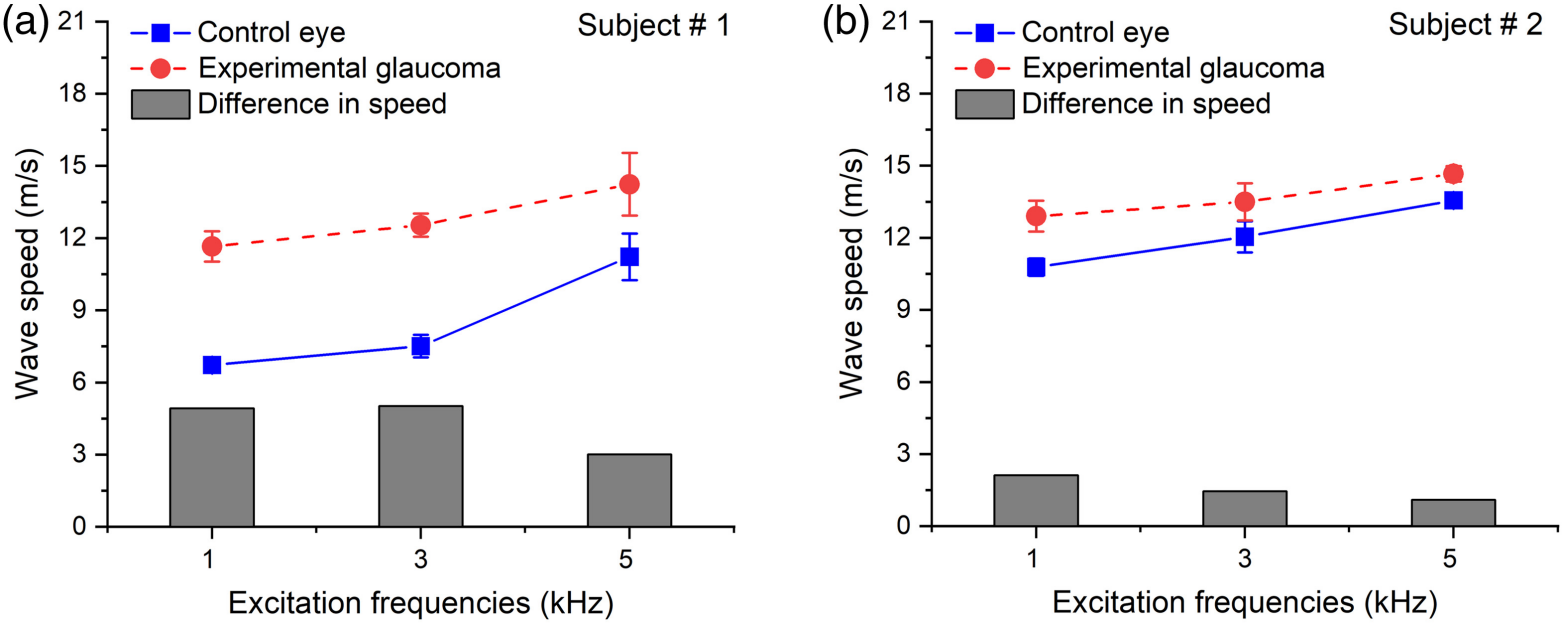
Average wave speed for (a) subject 1 and (b) subject 2 as a function of excitation frequency for the control and experimental glaucoma eye. Solid bars in panels (a)–(b) represent the difference in wave speed at each excitation frequency.

Figures 3(a) and 3(b) represent wave speed measurements for two NHP subjects at different excitation frequencies, respectively. In subject 1, the average wave speeds for the control eye were 6.7±0.3  m/s, 7.5±0.4  m/s, and 11.2±1.0  m/s at 1, 3, and 5 kHz, respectively. However, in the case of experimental glaucoma eye, the wave speed was significantly (U=0, Z=−2.19; p<0.01) higher at 11.7±0.6  m/s, 12.5±0.5  m/s, and 14.2±1.3  m/s at 1, 3, and 5 kHz, respectively. For subject 2, the average wave speeds for the control eye were 10.8±0.4  m/s, 12.0±0.6  m/s, and 13.6±0.2  m/s for 1, 3, and 5 kHz excitation, respectively. Just like subject 1, the treated eye showed a statistically significant (U=0, Z=−2.80; p<0.01) increase in wave speed of 12.9±0.6  m/s, 13.5±0.8  m/s, and 14.6±0.3  m/s at 1, 3, and 5 kHz excitation, respectively. In both subjects, we measured a significantly greater wave speed in the glaucomatous eye compared with the control eye at each frequency.

## 4 Discussion

In this work, we demonstrate to our knowledge the first study examining changes in the biomechanical properties of the cornea due to experimental glaucoma using OCE. Our results show that hypertension due to experimental glaucoma can cause an increase in corneal stiffness. This study does have certain limitations; measurements performed in only two animals restrict analysis of statistical significance, and although inducing ocular hypertension may be an effective glaucoma model, it may not be a perfect analogue for the disease. In addition, we have reported wave speed in this study, a semi-quantitative substitution for Young’s modulus. Future work will focus on analyzing elastic wave dispersion to translate the raw wave speed data to Young’s modulus using established mechanical models^42^ and more robust excitation, e.g., multifrequency,^24^^,^^27^ enabling accurate quantification of tissue stiffness accounting for sample geometry, boundary conditions, and intrinsic material properties such as density and Poisson’s ratio. Despite these limitations, our pilot study does suggest that OCE may be able to distinguish a difference in corneal stiffness due to remodeling of the cornea caused by ocular hypertension in POAG.

Previous works have shown that glaucoma may cause significant stiffening of the TM, and that ocular hypertension can cause significant strain and stiffening of the LC and ONH.^13^ As the structural composition of these tissues is similar to that of the cornea, it is possible that the mechanical and structural changes seen in the LC and the TM may also occur in the cornea. As the cornea is significantly more accessible, it could serve as a corollary for mechanical changes in the LC and TM in glaucoma.

Previous approaches to evaluate corneal stiffness are somewhat stymied by the impact of IOP changes on corneal rigidity,^43^^,^^44^ though it should be noted that some studies show a lack of correlation between IOP and OCE-measured corneal stiffness.^31^ Elevated IOP causing significant pressure against the cornea and can cause elevations in corneal stiffness, and alterations to corneal geometry due to ocular hypertension have been known to impact corneal biomechanical assessment.^45^ The expected IOP elevation due to glaucoma-induced hypertension was measured in these subjects, as shown in Table 1. However, the IOP measured prior to OCE was significantly less than the nominal IOP of the animal. This difference is due to the use of xylazine during the sedation procedure, an alpha-2-agonist that is commonly used in veterinary procedures as a muscle relaxant to aid sedation. Alpha-2-agonists have been shown to decrease IOP due to reduced production of aqueous humor.^40^ Although it is important to note that there was still a difference in IOP between control eyes and experimental glaucoma eyes, ΔIOP was much smaller due to the impact of xylazine. With IOP at reduced levels, we still noted an increase in corneal stiffness, which could suggest that corneal stiffness may have changed independent of IOP; potentially supporting the claim that prolonged ocular hypertension may cause ECM remodeling and a change in corneal stiffness. Further investigation with a larger cohort would be necessary to validate this claim.

## 5 Conclusion

In this work, we demonstrate the first use of OCE for measuring changes in corneal stiffness due to ocular hypertension analogous to glaucoma. For both subjects measured, we noted an increase in corneal stiffness in the eye with experimental glaucoma compared with the control. This pilot study suggests that prolonged ocular hypertension may cause structural remodeling of the cornea, increasing corneal stiffness independent of IOP. Future work will focus on expanding the study cohort and assessing changes in corneal stiffness in subjects as a function of time through longitudinal studies. The results of this work could guide future examinations of patients with glaucoma and aid in the early detection of this disease.

## Data Availability

The data that supports the findings of this article can be requested from the authors upon reasonable request. Due to the lack of established standards, public availability of the data and code will not provide any additional context for this publication.
